# Major depressive disorder and its association with adherence to antiretroviral therapy and quality of life: cross-sectional survey of people living with HIV/AIDS in Northwest Ethiopia

**DOI:** 10.1186/s12888-020-02865-w

**Published:** 2020-09-24

**Authors:** Biksegn Asrat, Crick Lund, Fentie Ambaw, Emily Claire Garman, Marguerite Schneider

**Affiliations:** 1 Alan J Flisher Centre for Public Mental Health, Department of Psychiatry and Mental Health, University of Cape Town, Cape Town, South Africa; 2 Centre for Global Mental Health, Department of Health Services and Population Research, King’s Global Health Institute, Institute of Psychiatry, Psychology and Neuroscience, King’s College London, London, UK; 3 School of Public Health, College of medicine and Health Sciences, Bahir Dar University, Bahir Dar, Ethiopia; 4 Department of Psychiatry, College of Health Sciences, Addis Ababa University, Addis Ababa, Ethiopia

**Keywords:** Major depressive disorder, HIV, Adherence to ART, Quality of life, Ethiopia

## Abstract

**Background:**

Major depression is believed to affect treatment adherence and overall quality of life (QoL) of people living with HIV/AIDS (PLWHA). Comorbid major depression contributes to a two-fold higher risk of mortality among PLWHA. Understanding the relationships of major depression, adherence to antiretroviral therapy (ART) and QoL is important to identify areas for intervention. The aim of this study is to examine relationship of major depressive disorder (MDD) and adherence to ART with QoL, and to investigate socio-demographic and clinical factors associated with MDD, adherence and QoL among PLWHA in Northwest Ethiopia.

**Method:**

A cross-sectional study was conducted in the ART clinic of Felege-Hiwot referral hospital in Northwest Ethiopia from July to October 2019. Adult PLWHA were selected using a systematic random sampling technique. Data were collected using interview administered questionnaires and chart reviews. Mini International Neuropsychiatric Interview and WHOQOL-HIV-BREF-Eth instruments were used to measure MDD and QoL respectively. Adherence to ART was assessed using pill count data from patients’ adherence monitoring chart. Univariate and multivariate Poisson regressions were used to assess associations of socio-demographic and clinical factors with MDD and adherence to ART. A multivariate linear regression was used to examine the associations of both MDD and adherence with overall QoL.

**Result:**

Of the total of 393 invited participants, 391 (99.5%) completed the interviews. MDD was negatively associated with overall QoL: participants with MDD had a lower QoL score of 0.17 points compared to those with no MDD. MDD was associated with reduced adherence to ART when functional disability was controlled (RR = 1.43; 95%CI = 1.05, 1.96; *p* = 0.025). However, there was no statistical association between adherence to ART and overall QoL. Functional disability was associated with both MDD (RR = 5.07; 95%CI = 3.27,7.86; *p* < 0.001) and overall QoL (β = 0.29; 95%CI = 0.21,0.36; p < 0.001).

**Conclusion:**

The relationship between MDD and QoL indicates the need for feasible, acceptable and evidence-based mental health interventions to reduce depression and improve overall QoL of PLWHA. We recommend future studies investigate causal relationships of MDD, adherence to ART and QoL of PLWHA to better understand priority areas for intervention.

## Background

Comorbid major depression affects the clinical outcomes of HIV/AIDS [1–3] and contributes to a two-fold higher risk of mortality [4]. Major depression has been one of the challenges of the 90–90-90 Joint United Nations Program on HIV/AIDS (UNAIDS) [4] that aimed to end the epidemic of HIV/AIDS in the world [5]. The sub-Saharan African (SSA) countries have been lagging far behind the target and comorbid major depression remains one of the challenges that has affected engagement with HIV care, leading to early loss to treatment and low viral suppression [4, 6].

Major depression is the most common mental disorder among people living with HIV/AIDS (PLWHA) and is estimated to be two to three times higher in PLWHA than in general population [4, 7–9]. However, prevalence reports are markedly variable across studies due partially to the use of different measurement approaches. A systematic review of studies from SSA found a range of 9 to 32% prevalence of probable depression [10]. A meta-analysis of studies from East Africa indicated a 38% pooled prevalence of probable depression [11]. A systematic review of studies from Ethiopia reported a 37% pooled prevalence of probable depression among PLWHA [12] which was much higher compared to the 11% pooled prevalence in the general population [13].

Major depression is prevalent in PLWHA due to their vulnerability to several factors. These factors can be classified as: 1) psychosocial factors such as HIV related stigma, disability and poverty [14–16]; 2) biological factors, such as structural and functional changes in the brain due to HIV infection (alteration in hypothalamic-pituitary-thyroid pathway) [9, 17–19] and due to chronic immune activation [2]; and 3) other comorbid conditions, including pre-existing comorbid illnesses other than HIV/AIDS which may increase risk of depression [18].

Major depression is believed to be one of the factors that affects adherence to ART although findings are inconclusive [20–22]. While many studies support the strong association, two systematic reviews did not find an independent relationship between major depression and adherence to ART [23, 24]. There are assumptions that cognitive impairment due to major depression, such as forgetfulness, poor concentration, impairment in memory, low executive functioning and difficulties with learning new behavior, may contribute to non-adherence to ART [25]. In addition, affective symptoms of major depression such as loss of interest, poor motivation, hopelessness and suicidality have also been reported to affect adherence to ART [4, 6].

Major depression is also an important determinant of Quality of Life (QoL) for PLWHA [6, 26–28]. QoL is a broad concept that includes several domains of functioning, namely physical, psychological, independence, social, environmental and spiritual functioning and can be affected by a variety of life circumstances [29–31]. However, the impact of major depression on poorer QoL outcomes seems to be high for PLWHA [32].

Although the pathways are not fully understood, major depression is reported to affect overall QoL of PLWHA either directly [3, 17, 20, 33] and/or indirectly by affecting adherence to antiretroviral therapy (ART) [19, 34]. Studies in HIV populations have indicated that major depression can affect several domains of QoL directly. For example, the effect of major depression on psychological and social functioning has been reported [35–37]. There are also studies that have shown a strong association between major depression and overall QoL, but the direction of association is unclear [32, 38].

Biological studies support the association of major depression with poor physical health and poor immune functioning [19, 39, 40]. For example, major depression has been reported to cause immunosuppression by altering the function of lymphocytes and decreasing natural killer cells [18]. The association of major depression with decreased CD4 cells, altered immune response and increased release of proinflammatory cytokines has been commonly reported [9, 19]. This may suggest that major depression can have a direct role in the physical deterioration and progress to advanced disease stage of PLWHA by interfering with immune functioning. Therefore, aiming to achieve overall improvement in QoL with the use of ART alone may not be successful without addressing major depression in PLWHA particularly in SSA [3, 19, 41].

In Ethiopia, several studies have examined prevalence of probable depression using screening tools [12]. However, diagnosis of major depression is important to develop treatment protocols, training manuals and to revise patient teaching guidelines. Furthermore, understanding relationships of major depression, adherence and QoL is important to design acceptable, feasible and evidence-based interventions for PLWHA. This study was a baseline survey used to identify and recruit cases with major depressive disorder (MDD) for a group interpersonal therapy intervention for PLWHA. This paper presents 1) the prevalence and associated factors of major depressive disorder (MDD), 2) the prevalence and associated factors of adherence to ART, 3) the factors associated with QoL, and 4) the associations of MDD and adherence to ART with overall QoL among PLWHA in Northwest Ethiopia.

## Method

### Setting and participants

This cross-sectional study was conducted in the ART clinic of Felege-Hiwot referral hospital (FHRH) in Northwest Ethiopia from 8th July to 7th October 2019. FHRH is one of the busiest hospitals in Northwest Ethiopia as described elsewhere [42] and serves a population of more than 7 million people. More than 3508 PLWHA had received ART treatment at the ART clinic over the 3 months preceding the data collection period, 2948 of which were adults 18 years and above. Sample size was calculated using the Select Statistical Services computer program using the following assumptions: a) a population proportion of 37% prevalence of depression which was taken from previous meta-analysis study findings done in people living with HIV/AIDS (PLWHA) in Ethiopia [1]. b) a 5% margin of error at 95% confidence interval. The sample size was estimated to be 357. Then we have added a 10% for the non-response rate which provided a total sample size of 392.7 (approximated to 393).

Participants were recruited using a systematic random sampling technique with an interval of seven. Patients medical cards were used to select every seventh client among those who were waiting for their ART follow-up at the ART clinic. Inclusion criteria were 18 years old and above, and able to provide informed consent. Exclusion criteria were being too ill to communicate or with a major physical illness, or emotional problems as result of an organic cause. In addition, those participants who diagnosed with cognitive deficits by clinicians were excluded from the study.

### Procedure

A research assistant approached every eligible client to explain the purpose of study, to ask for their willingness to be included in the study and to clarify the information in the informed consent sheet. Participants who signed consent forms were interviewed by trained data collectors in a private room in the ART clinic. The interviews were conducted in the local language (Amharic) and responses noted on paper questionnaires with no names and just a participant ID. The signed consent forms and completed questionnaires were kept in a locked file.

Data were collected using interview administered questionnaires and patient chart reviews. Socio-demographic, MDD, adherence, QoL, functioning and social support information was collected using an interviewer administered questionnaire. HIV related clinical information such as CD4 count, duration of ART treatment and ART regimen was collected from the patient charts. Field workers, including data collectors and supervisors, were trained for three days on participant selection, informed consent administration, data collection techniques and data handling.

Study participants who fulfilled the criteria for MDD or with suicidal idea were referred to group interpersonal therapy counselors for further evaluation and intervention. The counselors evaluated the severity and treatment need of each referred study participant and they intervened based on the given standard operating procedure. Those study participants who were severely sick were referred to the psychiatric clinic for advanced care and treatment.

### Measurements

#### MDD

The Mini International Neuropsychiatric Interview (MINI-7.0.2) a structured diagnostic assessment scale, was used to assess MDD [43]. The MINI-7.0.2 allows DSM-5 diagnosis and has been widely used as a gold standard measure of MDD [43, 44]. The MINI has been adapted to the Ethiopian context for use by non-specialized health professionals to detect MDD [45]. The diagnosis of MDD was made after organic causes were ruled out using the exclusion items in the MINI.

#### Quality of life (QoL)

The WHOQOL-HIV-BREF-Eth, that was adapted from the World Health Organization quality of life module and validated in the local language (Amharic) [46], was used to measure QoL in this study. The WHOQOL-HIV-BREF-Eth was validated for use in PLWHA in Ethiopia and has been used in several studies in HIV populations [29, 32, 38]. The validation shows that it has an excellent internal consistency with Cronbach alpha of 0.93. The instrument included two general items and six domains that give a total of 27 items. Study participants were asked to rate their subjective general wellbeing from 1 to 5 where low scores indicate low general wellbeing as perceived by study participants. Mean QoL score was calculated by dividing the sum scores of QoL by 27. Mean scores of each domain of QoL (physical, psychological, independence, social, environmental and spiritual) were also calculated independently.

#### Adherence to ART

We assessed adherence to ART using the pill count data, captured from patients’ re-fill chart and used for the purpose of clinical monitoring, together with clinicians’ report of skipped doses. Pill count is a valid method used to measure adherence to ART and has been used by several studies in resource limited settings [47, 48]. Upon examination of the frequencies and distribution of our data, we found that the data were skewed towards high adherence. Therefore, we narrowly defined *high adherence* as taking all doses of ART medications as prescribed by the clinician since the last appointment and *suboptimal adherence* was defined as skipping one or more doses of the prescribed medications.

#### Functional disability

The World Health Organization Disability Assessment Schedule (WHODAS-12) was used to measure functional disability. The WHODAS-12 instrument was high in acceptability, understandability and relevance to the WHODAS-36 instrument for the Ethiopian context [49]. Each item of the WHODAS-12 has Likert scale response options (No Difficulty = 0, Mild Difficulty = 1, Moderate Difficulty = 2, Severe Difficulty = 3, Extreme Difficulty/‘Cannot Do’ = 4). The scores of each item were summed and total scores can range from minimum of 0 to maximum of 48. In this study, functional disability was defined as a score of 36 or higher (i.e. having severe or extreme difficulty).

#### Perceived social support (PSS)

The Multidimensional Scale of Perceived Social Support (MSPSS) was used to evaluate perceived social support. The MSPSS has been adapted and validated in different settings across the world including in Africa [50–52]. The instrument has been shown to be reliable and valid for use in African cultures, for instance in Malawi [53] and Uganda [54]. The instrument evaluates perceived social support adequacy using three subscales: family, friends and significant others [50]. The MSPSS has 12-items (each subscale has 4-items) with 7-point Likert scale response options from 1 (very strongly disagree) to 7 (very strongly agree) [50, 55]. The value of each item was summed, and mean scores were calculated. The mean scores can range from minimum of 1 to maximum of 7. In this study mean scores ranging from 1 to 3 indicate low PSS, from 3 to 5 indicate moderate PSS and 5 to 7 indicate high PSS [55, 56].

### Data analysis

Data were entered into Epidata 4.6.0.0 [57] and analysed using SPSS statistics version 26 computer program [58]. Items of MDD, QoL, adherence to ART, PSS, functional disability and suicidality were computed to generate aggregated variables. Descriptive statistical analysis was used to summarize outcome variables, socio-demographic and clinical factors.

Univariate Poisson regression was used to identify association of each socio-demographic and clinical factor with MDD, and risk ratios are reported [59]. In multivariate Poisson regression, all factors with *p*-value < 0.2 in the univariate Poisson regression were entered in a multivariate analysis to control for confounding factors and generate adjusted risk ratios.

Similarly, we used univariate Poisson regression to identify the association of socio-demographic and clinical variables with adherence to ART, and risk ratios are reported. All factors with *p*-value of < 0.2 in the univariate Poisson regression were entered in a multivariate analysis to control for confounding factors and generate adjusted risk ratios.

We used linear regression to identify factors associated with the overall QoL. First, each of the socio-demographic and clinical variables were entered in a univariate model. Then, factors associated with overall QoL in the univariate analyses at a *p*-value < 0.2 were entered in a multivariate linear regression to adjust for confounding factors. These factors were also entered in a multivariate linear regression, for each domain of QoL separately, to examine the association of socio-demographic and clinical factors with each domain of QoL.

Finally, association of MDD and adherence to ART with overall QoL was examined using a generalized linear model. All socio-demographic and clinical factors associated with overall QoL at *p*-value of < 0.05 in the previous analysis were adjusted for in the model. An interaction analysis was also used in the model to assess interaction of MDD and adherence to ART on overall QoL.

### Ethics approval and consent to participate

This study was approved by the University of Cape Town’s Human Research Ethics Committee (HREC reference No. 653/2018). In addition, an approval letter was obtained from Bahir Dar University College of Medicine and Health Sciences’ Ethics Committee (reference No. 007/2018) and a permission letter was obtained from the Amhara Public Health Institute (APHI). All study participants signed a written informed consent. Illiterate study participants signed by fingerprint after a witness read the written informed consent.

## Results

### Socio-demographic and clinical characteristics of study participants

Of the total of 393 invited participants, 391 (99.5%) completed the interviews. More than two-thirds (69.3%, *n* = 269) of the study participants were women. The mean age of participants was 38.7 years old (SD = 9.1) ranging from 18 to 78 years old respectively. Three quarters (74.6%, *n* = 288) of the study participants were on first line ART treatments Table 1.

**Table 1:** Socio-demographic and clinical characteristics of the study participants, Felege-Hiwot Referral Hospital (FHRH), Northwest Ethiopia (*N* = 391)

Sociodemographic and clinical characteristics		Frequency	Percent
Age in years	18–29	51	13.2
	30–39	161	41.8
	40–49	119	30.9
	50+	54	14.0
Gender	Male	119	30.7
	Female	269	69.3
Marital status	Single	53	13.6
	Married/in relationship	187	47.8
	Divorced/widowed	151	38.6
Educational status	Illiterate	123	31.5
	Primary education	125	32.0
	Secondary education	93	23.8
	Tertiary education	50	12.8
Employment	Public servant	84	21.5
	Self employed	182	46.7
	None employed	124	31.8
Current CD4 count (last 6 months)	< 200	28	7.6
	200–349	69	18.6
	350–499	95	25.7
	> 500	178	48.1
Antiretroviral therapy regimen	First-line	288	74.6
	Second-line	98	25.4
Duration of ART treatment in years	1–5	72	19.0
	6–10	134	35.4
	11 and above	175	45.6
Major depressive disorder	Yes	127	32.5
	No	264	67.5
ART adherence	High adherence	289	81.9
	Low adherence	64	18.1

NOTE: First-line regimen is the use of first choice antiviral drugs and a switch to second-line regimen is recommended when there is treatment failure with first-line treatments

### Prevalence and associated factors of MDD

The overall prevalence of MDD was 32.5% (*n* = 127; 95%CI = 28.1,37.3) and it was 46.3% (*n* = 57) among illiterate, 44.4% (*n* = 55) among unemployed, 65.4% (*n* = 100) among functionally disabled study participants. The prevalence of MDD in each variable is presented in Table 2.

**Table 2:** Poisson regression: prevalence of and factors associated with major depressive disorder among people living with HIV/AIDS in Felege-Hiwot Referral Hospital (FHRH), Northwest Ethiopia, (*N* = 391)

Sociodemographic and clinical factors		Prevalence of MDD, % (n)	Unadjusted Poisson regression			Adjusted Poisson regression		
			Risk ratio	95%CI	***P*** value	Risk ratio	95%CI	***P*** value
Age in years ^a^	18–29	31.4 (16)	1.11	0.56, 2.19	0.770			
	30–39	33.5 (54)	1.18	0.69, 2.04	0.544			
	40–49	33.6 (40)	1.19	0.67, 2.09	0.555			
	50+	29.6 (16)	Reference	Reference				
Gender	Male	25.2 (30)	Reference	Reference				
	Female	36.1 (97)	1.47	0.97, 2.21	0.067	1.13	0.72, 1.78	0.600
Marital status	Married/in relationship	25.7 (48)	Reference	Reference		Reference	Reference	
	Single	37.7 (20)	1.47	0.87, 2.48	0.148	1.13	0.65, 1.96	0.675
	Divorced/widowed	39.1 (59)	1.52	1.04, 2.23	0.031	1.01	0.67, 1.53	0.968
Educational status	Illiterate	46.3 (57)	2.11	1.11, 4.02	0.024	1.04	0.43, 2.50	0.930
	Primary education	28.0 (35)	1.27	0.65, 2.51	0.485	0.90	0.39, 2.10	0.812
	Secondary education	25.8 (24)	1.17	0.58, 2.40	0.661	0.83	0.35, 1.99	0.682
	Tertiary education	22.0 (11)	Reference	Reference		Reference		
Employment	Public employed	17.9 (15)	Reference	Reference		Reference		
	Self employed	30.8 (56)	1.64	0.94, 2.85	0.083	1.40	0.68, 2.89	0.366
	Unemployed	44.4 (55)	2.36	1.35, 4.11	< 0.003	1.75	0.84, 3.63	0.135
Perceived social support	High support	24.3 (55)	Reference	Reference		Reference	Reference	
	Moderate support	41.9 (54)	1.70	1.17, 2.46	< 0.005	1.21	0.82, 1.78	0.337
	Low support	55.6 (15)	2.25	1.28, 3.97	< 0.005	1.25	0.67, 2.31	0.481
ART regimen	First-line	27.8 (80)	Reference	Reference		Reference	Reference	
	Second-line	45.9 (45)	1.64	1.14, 2.36	< 0.008	1.31	0.90, 1.91	0.157
Functional disability	No	11.0 (26)	Reference	Reference		Reference	Reference	
	Yes	65.4 (100)	5.76	3.77, 8.81	< 0.001	**5.07**	**3.27, 7.86**	**< 0.001**
Current CD4 count (last 6 months) ^a^	< 200	35.7 (10)	0.98	0.51, 1.91	0.969			
	200–349	26.1 (18)	0.72	0.43, 1.21	0.215			
	350–499	28.4 (27)	0.78	0.51, 1.22	0.285			
	> 500	36.5 (65)	Reference	Reference				
ART treatment duration in years ^a^	1–5	29.2 (21)	0.81	0.49, 1.32	0.387			
	6–10	29.1 (39)	0.80	0.54, 1.19	0.278			
	11 and above	36.4 (63)	Reference	Reference				

NOTE: First-line regimen is the use of first choice antiviral drugs and a switch to second-line regimen is recommended when there is treatment failure with first-line treatments. ART: antiretroviral therapy. ^a^ variables not included in multivariate Poisson regression

In the univariate regression (unadjusted analysis), being divorced or widowed, illiterate, unemployed, with low PSS, and on second line ART regimen were significantly associated with MDD. Results from the multivariate Poisson regression (adjusted analysis) show that only functional disability was associated with MDD: those who had functional disability (65.4%, *n* = 100) had 5.07 times the risk of MDD compared to those who had no functional disability (11%, *n* = 26) (95%CI = 3.27,7.86, *p* < 0.001).

### Prevalence of adherence to ART and associated factors

Overall, 81.9% of respondents (*n* = 289; 95%CI = 77.9, 85.8) had high adherence to ART (see Table 1). MDD was associated with reduced adherence to ART when functional disability was controlled for: those who had MDD (62.2%, *n* = 74) had 1.43 times the risk of suboptimal adherence to ART compared to those who had no MDD (91.9%, *n* = 215) (95%CI = 1.05, 1.96; *p* = 0.025) (see Table 3).

**Table 3:** Poisson regression: Prevalence of and factors associated with adherence to antiretroviral therapy among people living with HIV/AIDS in Felege-Hiwot Referral Hospital, Northwest Ethiopia, (*N* = 353)

Sociodemographic and clinical factors		Prevalence of high adherence, % (n)	Unadjusted Poisson regression			Adjusted Poisson regression		
			Risk ratio	95%CI	***P*** value	Risk ratio	95%CI	***P*** value
Age in years ^a^	18–29	79.2 (38)	Reference					
	30–39	82.4 (122)	1.04	0.73, 1.47	0.845			
	40–49	81.1 (86)	1.02	0.71, 1.47	0.920			
	50+	84.4 (38)	1.06	0.69, 1.64	0.792			
Gender ^a^	Male	83.3 (90)	1.03	0.80, 1.32	0.840			
	Female	81.0 (196)	Reference					
Marital status ^a^	Single	76.0 (38)	Reference					
	Married/ in relationship	85.7 (144)	1.13	0.79, 1.61	0.510			
	Divorced/widowed	79.3 (107)	1.04	0.72, 1.51	0.824			
Educational status ^a^	Illiterate	77.9 (88)	Reference					
	Primary education	79.5 (89)	1.02	0.76, 1.37	0.893			
	Secondary education	85.4 (70)	1.09	0.80, 1.50	0.566			
	Tertiary education	91.3 (42)	1.17	0.81, 1.69	0.396			
Employment ^a^	Public employed	91.9 (68)	1.21	0.88, 1.66	0.245			
	Self employed	81.3 (135)	1.07	0.82, 1.40	0.631			
	Unemployed	75.9 (85)	Reference					
Perceived social support ^a^	High support	82.8 (164)	1.14	0.74, 1.75	0.552			
	Moderate support	82.8 (101)	1.14	0.73, 1.78	0.568			
	Low support	69.2 (18)	Reference					
ART regimen ^a^	First-line	84.4 (217)	1.13	0.86, 1.47	0.383			
	Second-line	75.8 (69)	Reference					
Functional disability	No	89.6 (189)	1.27	0.99, 1.62	0.052	1.06	0.79, 1.41	0.710
	Yes	70.8 (98)	Reference			Reference		
Current CD4 count (last 6 months) ^a^	< 200	79.2 (19)	Reference					
	200–349	81.3 (52)	1.08	0.70, 1.68	0.719			
	350–499	86.0 (74)	1.15	0.76, 1.73	0.512			
	> 500	81.8 (130)	1.09	0.74, 1.60	0.658			
ART treatment duration in years ^a^	1–5	82.3 (51)	Reference					
	6–10	82.4 (98)	1.05	0.76, 1.45	0.765			
	11 and above	83.1 (133)	1.06	0.78, 1.44	0.709			
Major depressive disorder	No	91.9 (215)	1.48	1.14, 1.92	0.004	1.43	1.05, 1.96	0.025
	Yes	62.2 (74)	Reference			Reference		

NOTE: First-line regimen is the use of first choice antiviral drugs and a switch to second-line regimen is recommended when there is treatment failure with first-line treatments. ^a^ variables not included in multivariate Poisson regression; *ART* antiretroviral therapy

### Factors associated with QoL

The overall QoL mean score was 3.86 (SD = 0.42) and the mean scores for physical, psychological, independence, social, environmental and spiritual domains were 4.16 (SD = 0.56), 3.73 (SD = 0.65), 4.02 (SD = 0.56), 3.76 (SD = 0.60), 3.27 (SD = 0.61) and 4.42 (0.44) respectively. The multivariate linear regression shows that a higher level of education was positively associated with overall QoL: those with tertiary education had a higher QoL score of 0.34 compared to those who were illiterate (95%CI = 0.19,0.49; *p* < 0.001) (see Table 4). Taking first-line ART treatment was positively associated with overall QoL: participants who were taking first-line ART treatment had a higher QoL score of 0.12 compared to participants who were taking second-line ART treatment (95%CI = 0.03,0.20; *p* < 0.008). Similarly, taking ART for a longer duration was also positively associated with overall QoL: those who had taken ART for 6–10 years had a higher QoL score of 0.12 compared to those who had taken ART for less than 6 years (95%CI = 0.02,0.22; *p* = 0.022). High PSS was positively associated with overall QoL: participants who had high PSS had a higher QoL score of 0.43 compared to those who had low PSS (95%CI = 0.29,0.57; *p* < 0.001). Functional disability was also associated with overall QoL: those who had no functional disability had a higher QoL score of 0.29 compared to those who had functional disability (95%CI = 0.21,0.36; p < 0.001).

**Table 4:** Linear regression: Association of socio-demographic and clinical factors with overall quality of life among people living with HIV/AIDS in Felege-Hiwot Referral Hospital, Northwest Ethiopia, (*N* = 391)

Factors		Mean (SD)	Unadjusted linear regression			Adjusted multivariate linear regression		
			Coefficient	95%CI	***P*** value	Coefficient	95%CI	***P*** value
Age in years	18–29	3.76 (0.40)	Reference	Reference		Reference	Reference	
	30–39	3.86 (0.44)	0.27	−0.26, 0.80	0.313	0.07	−0.04, 0.19	0.209
	40–49	3.91 (0.43)	0.49	−0.07, 1.05	0.087	0.10	−0.2, 0.23	0.102
	50+	3.83 (0.37)	0.18	− 0.48, 0.83	0.601	0.06	−0.08.0.21	0.371
Gender ^a^	Male	3.90 (0.39)	0.23	−0.16, 0.61	0.255			
	Female	3.84 (0.44)	Reference	Reference				
Marital status	Single	3.77 (0.45)	−0.08	− 0.65, 0.48	0.770	− 0.04	− 0.16, 0.08	0.530
	Married/ in relationship	3.93 (0.39)	0.57	0.18, 0.95	< 0.004	−0.01	− 0.08, 0.08	0.974
	Divorced/widowed	3.79 (0.45)	Reference	Reference		Reference	Reference	
Educational status	Illiterate	3.67 (0.50)	Reference	Reference		Reference	Reference	
	Primary education	3.87 (0.37)	0.78	0.35, 1.21	< 0.001	**0.13**	**0.03, 0.22**	**< 0.008**
	Secondary education	3.95 (0.35)	1.12	0.65, 1.58	< 0.001	**0.20**	**0.09, 0.31**	**< 0.001**
	Tertiary education	4.09 (0.31)	1.69	1.13, 2.25	< 0.001	**0.34**	**0.19, 0.49**	**< 0.001**
Employment status	Public employed	4.01 (0.32)	0.85	0.37, 1.33	0.001	−0.07	−0.19, 0.05	0.274
	Self employed	3.83 (0.42)	0.14	−0.27, 0.55	0.499	0.01	−0.08, 0.09	0.886
	None employed	3.81 (0.44)	Reference	Reference		Reference	Reference	
ART regimen	First-line	3.90 (0.41)	0.59	0.18, 0.99	< 0.005	**0.12**	**0.03, 0.20**	**< 0.008**
	Second-line	3.76 (0.45)	Reference	Reference		Reference	Reference	
Current CD4 count (last 6 months)	< 200	3.85 (0.45)	Reference	Reference		Reference	Reference	
	200–349	3.88 (0.37)	0.34	−0.315, 0.99	0.307	−0.01	− 0.14, 0.13	0.937
	350–499	3.94 (0.38)	0.58	−0.042, 1.20	0.067	0.06	−0.07, 0.19	0.343
	> 500	3.83 (0.43)	0.16	−0.40, 0.72	0.575	0.01	−0.11, 0.13	0.865
Duration of ART treatment in years	1–5	3.83 (0.49)	Reference			Reference	Reference	
	6–10	3.91 (0.41)	0.35	−0.14, 0.85	0.157	**0.12**	**0.02, 0.22**	**0.022**
	11 and above	3.84 (0.41)	0.05	−0.42, 0.52	0.835	−0.01	− 0.11, 0.10	0.931
Perceived social support	Low support	3.38 (0.62)	Reference	Reference		Reference	Reference	
	Moderate support	3.68 (0.36)	0.90	0.27, 1.54	0.006	**0.18**	**0.04, 0.33**	**0.014**
	High support	3.86 (0.42)	2.19	1.59, 2.80	< 0.001	**0.43**	**0.29, 0.57**	**< 0.001**
Functional disability	No	4.01 (0.33)	1.42	1.08, 1.75	< 0.001	**0.29**	**0.21, 0.36**	**< 0.001**
	Yes	3.64 (0.47)	Reference	Reference		Reference	Reference	

NOTE: First-line regimen is the use of first choice antiviral drugs and a switch to second-line regimen is recommended when there is treatment failure with first-line treatments. ^a^ not included in the final model of analysis; *ART* antiretroviral therapy

In addition, the adjusted linear regressions by domains of QoL show that age and employment were statistically associated with one or more domains of QoL (see Table 5). In the univariate analysis, being married or in a relationship and public employment were significantly associated with a higher QoL score.

**Table 5:** Linear regression: association of socio-demographic and clinical factors with the domains of quality of life among people living with HIV/AIDS in Felege-Hiwot Referral Hospital, Northwest, Ethiopia, (*N* = 391)

Sociodemographic and clinical factors		Physical β (95%CI)	Psychological β (95%CI)	Independence β (95%CI)	Social β (95%CI)	Environmental β (95%CI)	Spiritual β (95%CI)
Age	18–29	Reference	Reference	Reference	Reference	Reference	Reference
	30–39	−0.05 (− 0.22, 0.11)	0.07 (− 0.09, 0.23)	− 0.01 (− 0.16, 0.15)	0.12 (− 0.03, 0.28)	**0.18 (0.01, 0.37)**	0.07 (− 0.07, 0.20)
	40–49	− 0.10 (− 0.27, 0.08)	0.10 (− 0.07, 0.27)	−0.03 (− 0.20, 0.14)	0.10 (0.07, 0.27)	**0.26 (0.06, 0.46)**	0.08 (− 0.06, 0.23)
	50+	−0.11 (− 0.32, 0.09)	0.16 (− 0.05, 0.36)	− 0.09 (− 0.30, 0.09)	0.14 (− 0.05, 0.33)	**0.23 (0.02, 0.46)**	0.11 (− 0.05, 0.28)
Marital status	Married/relationship	Reference	Reference	Reference	Reference	Reference	Reference
	Single	−0.10 (− 0.27, 0.07)	0.01 (− 0.16, 0.17)	− 0.09 (− 0.25, 0.07)	−0.16 (− 0.32, 0.01)	0.18 (− 0.02, 0.37)	− 0.09 (− 0.23, 0.05)
	Divorced/ widowed	0.01 (− 0.10, 0.13)	0.07 (− 0.04, 0.18)	0.06 (− 0.05, 0.17)	−0.08 (− 0.19, 0.03)	0.07 (− 0.07, 0.20)	−0.05 (− 0.14, 0.05)
Educational status	Illiterate	Reference	Reference	Reference	Reference	Reference	Reference
	Primary education	**0.20 (0.07, 0.33)**	**0.17 (0.04, 0.30)**	**0.22 (0.09, 0.35)**	0.11 (− 0.01, 0.24)	0.12 (− 0.03, 0.28)	**0.12 (0.01, 0.22)**
	Secondary education	**0.21 (0.06, 0.35)**	**0.22 (0.07, 0.36)**	**0.21 (0.07, 0.35)**	0.13 (−0.01, 0.27)	**0.31 (0.14, 0.48)**	**0.13 (0.01, 0.25)**
	Tertiary education	**0.27 (0.06, 0.48)**	**0.51 (0.30, 0.72)**	**0.35 (0.15, 0.56)**	0.16 (−0.04, 0.37)	**0.33 (0.10, 0.57)**	**0.23 (0.06, 0.41)**
Employment status	Public servant	−0.08 (− 0.26, 0.09)	−0.09 (− 0.26, 0.09)	0.01 (− 0.16, 0.18)	−0.01 (− 0.17, 0.16)	−0.04 (− 0.23, 0.16)	−0.09 (− 0.23, 0.06)
	Self employed	−0.04 (− 0.16, 0.08)	**0.17 (0.05, 0.29)**	0.07 (− 0.04, 0.19)	−0.11 (− 0.22, 0.01)	−0.01 (− 0.14, 0.13)	−0.07 (− 0.17, 0.02)
	Unemployed	Reference	Reference	Reference	Reference	Reference	Reference
Perceived social support	Low support	Reference	Reference	Reference	Reference	Reference	Reference
	Moderate support	0.15 (−0.05, 0.34)	**0.34 (0.15, 0.53)**	**0.23 (0.05, 0.42)**	0.10 (− 0.08, 0.29)	0.11 (−0.13, 0.34)	**0.19 (0.03, 0.35)**
	High support	**0.25 (0.06, 0.44)**	**0.60 (0.42, 0.79)**	**0.37 (0.19, 0.56)**	**0.68 (0.50, 0.87)**	**0.45 (0.22, 0.68)**	**0.28 (0.13, 0.44)**
ART regimen	First-line	**0.15 (0.04, 0.27)**	**0.19 (0.07, 0.30)**	**0.11 (0.01, 0.23)**	0.10 (−0.01, 0.21)	0.08 (−0.05, 0.22)	0.08 (− 0.02, 0.17)
	Second-line	Reference	Reference	Reference	Reference	Reference	Reference
Functional Disability	No	**0.39 (0.28, 0.49)**	**0.55 (0.44, 0.66)**	**0.35 (0.25, 0.45)**	**0.25 (0.15, 0.35)**	0.10 (−0.02, 0.22)	**0.17 (0.08, 0.26)**
	Yes	Reference	Reference	Reference	Reference	Reference	Reference
Current CD4 count (last 6 months)	< 200	Reference	Reference	Reference	Reference	Reference	Reference
	200–349	−0.07 (−0.27, 0.12)	0.07 (−0.12, 0.26)	− 0.03 (− 0.21, 0.16)	0.04 (− 0.14, 0.22)	−0.08 (− 0.30, 0.15)	0.14 (− 0.02, 0.30)
	350–499	0.03 (− 0.16, 0.21)	0.08 (− 0.10, 0.26)	0.07 (− 0.10, 0.25)	0.04 (− 0.14, 0.21)	−0.03 (− 0.24, 0.18)	0.13 (− 0.02, 0.28)
	> 500	0.01 (− 0.17, 0.17)	0.01 (− 0.15, 0.18)	−0.04 (− 0.20, 0.12)	0.03 (− 0.13, 0.19)	−0.05 (− 0.24, 0.15)	0.05 (− 0.09, 0.19)
ART treatment duration in years	1–5	Reference	Reference	Reference	Reference	Reference	Reference
	6–10	**0.17 (0.03, 0.32)**	0.12 (−0.02, 0.27)	0.13 (−0.01, 0.27)	0.05 (− 0.09, 0.19)	0.01 (− 0.15, 0.18)	**0.17 (0.05, 0.29)**
	11+	0.03 (−0.11, 0.18)	0.03 (−0.12, 0.17)	− 0.05 (− 0.19, 0.09)	−0.04 (− 0.09, 0.19)	−0.14 (− 0.30, 0.03)	0.04 (− 0.08, 0.16)

The results in this table were identified in multivariate linear regression. All variables associated with overall QoL at > 0.2 were controlled multivariate analyses with each domain of QoL. First-line regimen is the use of first choice antiviral drugs and a switch to second-line regimen is recommended when there is treatment failure with first-line treatments. *ART* antiretroviral therapy

### Association of MDD and adherence to ART with overall QOL

After controlling for socio-demographic and clinical factors associated with overall QoL, results of the multivariate linear regression show that MDD was negatively associated with overall QoL: participants with MDD had a lower QoL score of 0.06 compared to those with no MDD (95%CI = 0.89, 0.99; *p* < 0.047) (see Table 6). However, there was no statistical association between adherence to ART and overall QOL. The generalized linear model presented no interaction effect of MDD and adherence to ART on QoL scores (95%CI = 0.94,1.07; β = 0.01; *p* = 0.866).

**Table 6:** Generalised linear model univariate analysis: association of major depressive disorder and adherence to antiretroviral therapy with the overall quality of life after adjusting for socio-demographic and clinical factors among people living with HIV/AIDS in Felege-Hiwot Referral Hospital, Northwest Ethiopia, *N* = 391

Covariates		Mean (SD)	Adjusted linear regression		
			Coefficient	95%CI	***P*** value
Major depressive disorder	No	3.99 (0.33)	Reference	**Reference**	
	Yes	3.59 (0.47)	**−0.06**	**0.89, 0.99**	**0.047**
Adherence to ART	High adherence	3.86 (0.40)	Reference		
	Low adherence	3.73 (0.46)	−0.03	0.93, 1.02	0.277
MDD*Adherence to ART			0.01	0.94, 1.07	0.866

NOTE: All variables associated with overall QoL at > 0.05 were controlled for in this model (age, marital status, educational status, employment status, antiretroviral therapy (ART) regimen, CD4 count, duration of ART treatment, perceived social support and functional disability). ART: antiretroviral therapy

## Discussion

Unlike most of the previous studies in HIV populations in Ethiopia, this study examined the prevalence of major depression using a diagnostic instrument. We found that one third of the study participants had MDD, and that being divorced or widowed, illiterate, unemployed, with low perceived social support, and on second line ART regimen were significantly associated with MDD in univariate regressions (unadjusted analysis). MDD was strongly associated with functional disability and suboptimal adherence to ART in the multivariate regression (adjusted analysis). We also identified associations between higher overall QoL and better educational status, better PSS, functioning, taking ART for a longer duration and taking first-line ART treatments. Our study indicated that MDD was strongly associated with lower overall QoL.

More than two thirds of the study participants were women. However, there was no difference in prevalence of MDD between men and women. The reason for the majority of the participants being women could be due to a higher prevalence of HIV/AIDS among women than men. This is supported by several reports that have shown that HIV/AIDS is more prevalent in women than men in Ethiopia [60]. Our study found that there was no difference in prevalence of MDD by age and gender which is in agreement with findings from another study reporting that MDD is prevalent in all age groups and in both genders of PLWHA [10]. Biological impact of HIV [61], stigma and poor social support [8], and low socioeconomic conditions [10] all contribute to a high prevalence of MDD among PLWHA.

In this study, there was a significant association between PSS and MDD in the univariate regression but no association between PSS and MDD in the multivariate regression. This finding is inconsistent with a systematic review of longitudinal studies that reported poorer PSS causes worse outcomes of MDD in terms of functioning and recovery [62] and with another systematic review of studies from Africa that reported presence of a strong relationship between PSS and MDD [8]. However, a systematic review of studies from high income countries [63] and another longitudinal study among adolescents from China [64] reported no relationship between level of PSS and MDD. There is conflicting global evidence on a causal relationship between PSS and MDD. Interpersonal theories of depression indicate that poor social support leads to onset of MDD [65]. In contrast, a more recent longitudinal study presented evidence that PSS is a consequence of MDD contrary to social causation theories of depression [64], reporting that the negative views of people with MDD can make it uncomfortable for peers to provide social support. In addition, lack of social skills of people with MDD to create and maintain a relationship may result in a social avoidance state. Despite our findings, we believe that social support could be an important intervention for MDD. MDD caused by a psychosocial crisis can be effectively treated by strong social support, which helps to decrease interpersonal stress and improve interpersonal skills [65].

Our study shows that functional disability was independently associated with MDD. Functional disability prevents people with MDD from performing daily activities, leads to loss of social roles and lack of access to resources for basic needs [66]. Failure to fulfil self needs and social responsibilities can lead to onset of full blown MDD, or reciprocally MDD can lead to functional disability [67]. Furthermore, people with functional disability need more intensive social and material support due to poverty and this could aggravate pre-existing MDD [68].

Our finding about the association of several socio-demographic and clinical factors with overall QoL is consistent with previous findings. Better educational status [69–71], being employed [72, 73], good social support [69, 72], functioning [20] and access to first line ART treatment [71, 74] have been identified as predictors of improved QoL. Our findings support the hypothesis that PLWHA with better education and with good physical functioning could have better income and employment opportunities to gain better QoL.

None of the socio-demographic and clinical factors showed statistical associations with adherence to ART which is inconsistent with previous findings [24]. This can be explained by challenges in measuring adherence to ART using pill count. There was also inconsistency in using specific measures across studies that may be misleading in assessing treatment adherence in HIV populations. But MDD seems an important predictor of suboptimal adherence to ART which is supported by several findings from previous studies [71, 72]. This could be due to MDD related cognitive impairment, such as memory loss and forgetfulness in taking ART medications [73–75], or due to poor motivation, hopelessness and wishing to die [4]. Our findings show the need to integrate mental health interventions with existing HIV care as MDD has been found to be one of the major barriers to the global agenda to end the HIV epidemic specially in SSA countries [4–6].

The observed relationship between MDD and overall QoL was consistent with previous findings in HIV populations [69, 71–73]. Evidence shows that MDD consistently leads to worse health outcomes in PLHWA not solely due to difference in adherence to ART but may also be due to direct effect of MDD on physiological functioning [1]. Firstly, MDD can affect QoL by interfering with immune system functioning. Secondly, MDD affects positive thoughts for change that can lead to lower adherence to ART. Thirdly, the relationship between MDD and poor QoL can also explained by indirect effects of MDD on QoL such as financial insecurity, unemployment and financial dependency on others [8]. The overall findings of this study indicated that addressing MDD should be a priority to facilitate recovery and functioning of PLWHA. A recent systematic review and meta-analysis of RCTs found that psychological treatments enhance immune system functioning and are viable for improving immune function [75]. Therefore, we recommend the use of psychological treatments for treatment of depressive symptoms and to improve immune functioning that could help to improve overall QoL of PLWHA. We recommend that mental health researchers develop evidence-based psychological treatments that are appropriate for PLWHA to manage depression, and to improve adherence to ART and overall quality of life of PLWHA. Furthermore, we believe that this study could have several implications for health care providers and policy makers to consider mental health conditions as a priority and to act accordingly. Specifically, routine screening services for depression and other mental disorders should be established at ART clinics and mental health services integrated with HIV care services in Ethiopia.

A major strength of this study was the use of instruments validated in Ethiopia. However, it has also several limitations. Firstly, it is a cross-sectional study that doesn’t allow us to identify causal relationships between independent and outcome variables. Secondly, the use of a self-report measure to assess adherence to ART may not be the right approach and future studies should consider alternative approaches. Thirdly, readers should note that *suboptimal adherence to ART* was defined narrowly as skipping one or more doses of ART medications. Fourth, there may have been recall bias in the manner in which study participants shared their information.

## Conclusion

This study indicates that both MDD and QoL have a strong relationship with functional disability among PLWHA in Northwest Ethiopia. The strong relationship between MDD and QoL indicates the need to integrate feasible, acceptable and evidence-based mental health interventions within the existing HIV care services to improve the overall QoL of PLWHA. We recommend future studies to investigate causal relationships of MDD, adherence to ART and QoL of PLWHA to better understand priority areas for intervention.

## Data Availability

Not applicable.

## Abbreviations

ART: Antiretroviral therapy
MDD: Major depressive disorder
PLWHA: People living with HIV/AIDS
QoL: Quality of life
